# Decoding eQTLs: unraveling their role in spondyloarthropathies pathogenesis and potential therapeutics

**DOI:** 10.3389/fimmu.2025.1624263

**Published:** 2025-08-27

**Authors:** Nabaa Ihsan Awadh, Asal Adnan Ridha, Marwah Muayad Younus, Yasameen Abbas Humadi, Faiq I. Gorial, Khalid Burhan Khalid, Yousif Alaa Rasool

**Affiliations:** ^1^ Rheumatology Unit, Department of Internal Medicine, College of Medicine, University of Baghdad, Baghdad, Iraq; ^2^ Department of Medicine- Ibn Sina Medical College, Baghdad, Iraq; ^3^ Department of Internal Medicine- Alnahrain University- College of Medicine, Baghdad, Iraq; ^4^ Oral Surgery Unit, College of Dentistry, Dijlah University, Baghdad, Iraq; ^5^ College of Medicine, University of Baghdad, Baghdad, Iraq

**Keywords:** eQTLs, spondyloarthropathies, pathogenesis, therapeutics, genetic regulation, inflammation

## Abstract

Expression quantitative trait loci (eQTLs) represent genetic variants that influence gene expression, providing insights into the mechanisms linking genetic predisposition to complex diseases, including spondyloarthropathies (SpAs). SpAs, encompassing ankylosing spondylitis, psoriatic arthritis, reactive arthritis, and enteropathic arthritis, are related heterogeneous conditions with strong genetic components, particularly in HLA-B27 and non-HLA loci, but phenotypically distinct disorders. Recent advances in genome-wide association studies (GWAS) and transcriptomic studies have shown the potential of such eQTLs to provide insights into the disease biology, delineate potential drug targets, and enable precision medicine. This mini-review provides a comprehensive overview of eQTLs—discovery, definition, and functional implication into eQTLs gene regulation. We review their role in SpA pathogenesis, controversy and methodological issues in eQTL studies, literature gaps, and directions for the future. By combining genetic, immunological, and computational information, we aim to present an unbiased perspective on the role of eQTLs in advancing SpA research and treatment.

## 1 Introduction

The identification of expression quantitative trait loci (eQTLs) has transformed our views of the genetic architecture of complex disorders. EQTLs act as mediators of genetic variation, extending beyond the genomic origin to phenotypic traits, particularly through the regulation of gene expression. This is especially important in immune-mediated disorders, such as spondyloarthropathies (SpAs), in which genetic predisposition plays a key role. SpAs are a group of inflammatory rheumatic diseases characterized by axial and peripheral joint involvement, enthesitis, and extraarticular manifestations (1, 2). However, despite many advances, genetic evidence has not yet converted itself into practical knowledge and thus therapies. This mini-review captures recent advances in the understanding of eQTLs in SpA, focusing on both achievements and limitations in the field.

## 2 Methodology: literature search and selection strategy

A comprehensive narrative review methodology was employed to gather relevant literature addressing expression quantitative trait loci (eQTLs) and their role in the pathogenesis of spondyloarthropathies (SpA). The search strategy covered publications available up to May 2025 to capture both foundational and contemporary research developments in the field.

Electronic databases including PubMed (using MeSH terms), Scopus, and Web of Science (using free-text keyword searches) were systematically queried. The search terms included combinations of: “eQTL”, “expression quantitative trait loci”, “genetic regulation”, “spondyloarthropathies”, “ankylosing spondylitis”, “psoriatic arthritis”, and “IL-23/IL-17 axis”.

This search was supplemented by manual screening of reference lists from relevant articles to identify additional pertinent publications. Inclusion criteria comprised studies reporting on eQTL discovery methods, functional characterization, tissue- and cell-specific effects, and their immunogenetic or translational implications in SpA. Exclusion criteria included articles lacking methodological detail, and case reports.

All selected studies were critically appraised for methodological rigor, sample size adequacy, relevance to SpA pathogenesis, and integration of eQTL data with immunogenetic or multi-omics findings. The final selection was synthesized to present a comprehensive, balanced overview of current evidence and to identify gaps requiring further investigation.

## 3 Fundamentals of eQTLs

### 3.1 Definition and types of eQTLs

Expression quantitative trait loci (eQTLs) are genomic loci that regulate gene expression either in cis (proximal to the gene) or in trans (distal to the gene) (3). Cis-eQTLs typically influence gene expression by directly affecting regulatory elements such as promoters and enhancers located near the gene. In contrast, trans-eQTLs exert their effects indirectly by modulating upstream regulators—transcription factors, signaling pathways, chromatin-modifying proteins, or RNA regulators—which in turn influence the expression of distant genes across different chromosomal regions. This occurs through complex regulatory networks that coordinate gene expression at a systems-level (e.g., through co-expressed gene modules or master regulatory hubs). Recognizing these distinctions is important for understanding their respective contributions to disease mechanisms (4).

### 3.2 Discovery methods

High-throughput sequencing-based technologies (e.g., RNA-seq and GWAS) have allowed the discovery of eQTLs (5). Integrating methods based on GWAS and eQTL data is particularly powerful for identifying disease-contributing loci (6). With increasingly complex data sets, advanced computational resources, including eQTL mapping and colocalization analyses, enhance our ability to make sense of these sophisticated data (7).

### 3.3 Functional characterization

Functional assays, such as chromatin immunoprecipitation sequencing (ChIP-seq) and CRISPR-based perturbations, are paramount for demonstrating eQTL modulatory effects and for elucidating the mechanisms they regulate (8). These methods can be used in the discovery of causal variants and of potentially subsequent biological effects.

## 4 Spondyloarthropathies: genetic and molecular landscape

### 4.1 Overview of SpA

Spondyloarthropathies (SpAs) are a group of chronic inflammatory diseases, including ankylosing spondylitis (AS), psoriatic arthritis (PsA), reactive arthritis, and enteropathic arthritis. Such conditions share important clinical features, including enthesitis (inflammation at tendon and ligament attachment sites), axial skeletal involvement, and extra-articular manifestations including uveitis and inflammatory bowel disease. The shared clinical features indicate a common genetic and immunological basis of these diseases (9).

### 4.2 Genetic basis of SpA

#### 4.2.1 HLA-B27 and beyond

**Figure 1:**
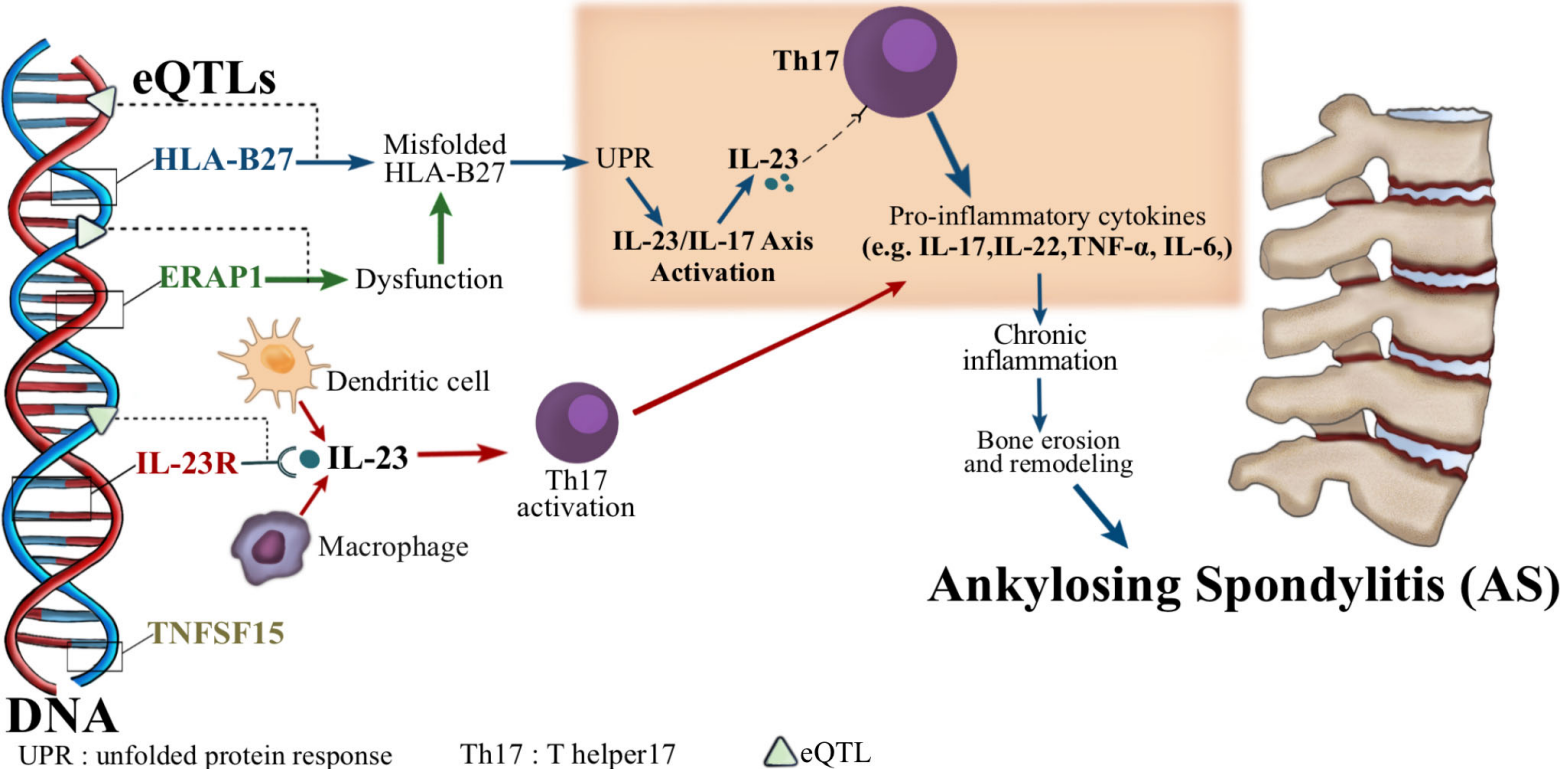
The role of eQTLs and cytokine pathways in the pathogenesis of ankylosing spondylitis (AS). This diagram illustrates how expression quantitative trait loci (eQTLs) affect key genes (HLA-B27, ERAP1, IL-23R, TNFSF15) associated with AS. Misfolding of HLA-B27 proteins and ERAP1 dysfunction both lead to endoplasmic reticulum (ER) stress and activation of the unfolded protein response (UPR), which in turn activates the IL-23/IL-17 axis. This activation leads to increased production of IL-23, which binds to Th17 cells and promotes their activation. Activated Th17 cells secrete pro-inflammatory cytokines such as IL-17, IL-22, TNF-α, and IL-6, leading to chronic inflammation, bone erosion, and remodeling. Ultimately, this process contributes to the development of ankylosing spondylitis.

The HLA-B27 gene is the most significant genetic associate of SpA, with up to 90% of ankylosing spondylitis patients harboring the gene. Its pathogenic effects involve antigen presentation, misfolding-mediated endoplasmic reticulum (ER) stress, and formation of arthritogenic HLA-B27 homodimers (10). SpA’s genetic architecture further includes additional susceptibility loci such as ERAP1, IL23R, and TNFSF15. These genes collectively underscore the polygenic nature of SpA, emphasizing pathways involved in antigen processing and presentation (e.g., peptide trimming by ERAP1 and loading onto HLA-B27), as well as key inflammatory processes like IL-23/IL-17 axis activation (**Figure 1**) (11–14).

#### 4.2.2 GWAS findings

Recent genome-wide association studies (GWAS) have identified over 100 genetic loci associated with spondyloarthropathies, many of which are situated in non-coding regions with regulatory potential distributed across the genome (15, 16). It is understood these are key regulatory areas that play a role in gene expression rather than direct protein production. Methods such as eQTL mapping have been highly useful in interpreting these GWAS findings by connecting non-coding variants and gene expression changes. Such insights are really crucial in the identification of causal pathways, as well as potential therapeutic targets, and stress the necessity to marry molecular and cellular studies with the datasets (17).

## 5 eQTLs in SpA: mechanisms and implications

### 5.1 Linking GWAS to functional biology

#### 5.1.1 eQTLs and immune regulation

The interleukin-23 (IL-23)/interleukin-17 (IL-17) axis represents a pivotal immunopathogenic pathway in the development and perpetuation of spondyloarthropathies (SpA). IL-23, a heterodimeric cytokine produced predominantly by dendritic cells and macrophages, plays a central role in the maintenance and expansion of T helper 17 (Th17) cells. Th17 cells, through the secretion of IL-17A, IL-17F, and other pro-inflammatory cytokines, orchestrate the recruitment of neutrophils and the activation of stromal cells at entheses, synovial tissues, and axial skeletal structures — the characteristic sites of SpA pathology (18).

Genetic studies, particularly genome-wide association studies (GWAS), have robustly implicated polymorphisms within genes regulating this pathway, notably IL23R and TYK2, both of which modulate Th17 cell biology. IL23R encodes the receptor subunit specific for IL-23, while TYK2 encodes a tyrosine kinase essential for downstream signal transduction following IL-23 engagement. Variants in these genes not only confer susceptibility to SpA but also influence gene expression patterns, as evidenced by expression quantitative trait loci (eQTL) analyses, further supporting their functional relevance (12, 17).

In this context, several eQTLs have been identified that modulate IL23R expression in immune cell subsets, particularly in CD4+ T cells, contributing to dysregulated IL-23-mediated signaling in genetically predisposed individuals (19). Similarly, TYK2 eQTLs have been shown to influence cytokine signaling thresholds, impacting Th17 differentiation and effector function (20). These findings underscore the biological plausibility of targeting the IL-23/IL-17 axis in SpA, which has been clinically validated through the success of IL-17A and IL-23 inhibitors in managing axial and peripheral disease manifestations (21).

#### 5.1.2 Tissue-specific eQTL effects

eQTL effects are known to vary across tissues and cell types due to differences in chromatin landscape and transcription factor binding (22). In the context of SpA, while data on synovial or entheseal fibroblast eQTLs are not yet available, robust evidence exists for cell-type–specific eQTLs in immune cells such as T cells and monocytes. These immune cell–specific regulatory effects implicate key SpA-associated genes including IL23R, ERAP1, TYK2, RUNX3, and B3GNT2 [19, 20].

For example, Kim-Hellmuth et al. (2020) identified cis-eQTLs for IL23R and TYK2 that are active in CD4+ T cells but absent in other tissues, demonstrating the importance of immune context in genetic regulation (19). Similarly, van der Wijst et al. used single-cell RNA sequencing to reveal cell-type–specific eQTLs, underlining that eQTL signals show heterogeneity across immune subsets (20).

This underscores the necessity of mapping eQTLs in Synovium and entheses, where disease phenotypes manifest, because these effects may be tissue-specific and invisible in blood-based analyses. We encourage future studies to apply single-cell or organ-specific approaches to determine the regulatory activity of SpA-associated loci in these target tissues.

### 5.2 Key eQTLs in SpA

#### 5.2.1 ERAP1 eQTLs

ERAP1 eQTLs modulate antigen processing and presentation, thereby modulating HLA-B27 stability and folding. ERAP1 variants affect enzymatic activity, thereby altering peptide trimming and consequently immune responses (23).

#### 5.2.2 IL23R eQTLs

Variants influencing IL23R expression alter responses of Th17 cells, which are the hallmark of SpA pathogenesis. Targeting IL23R-related pathways represents a promising therapeutic strategy (14).

A summary of the strongest reported QTLs and eQTLs associated with SpA, including their chromosomal locations, associated SNPs, immunological pathways, and cell-specific expression effects, is provided in **Table 1**.

**Table 1:** Comparison of eQTLs and QTLs in Spondyloarthropathy-Associated Genes.

Category	QTLS	eQTLs
Example Genes	HLA-B27, ERAP1, IL23R, TNFSF15 (11, 14)	RUNX3, TNFRSF1A, IL6R (19)
Chromosomal Regions	6p21 (HLA-B27), 5q15 (ERAP1), 1p31 (IL23R) (11, 12)	1p36 (RUNX3), 12p13 (TNFRSF1A), 1q21 (IL6R) (19)
Associated SNPs	rs30187 (ERAP1), rs11209026 (IL23R) (12)	rs4648889 (RUNX3), rs1800693 (TNFRSF1A), rs4129267 (IL6R) (19)
Trait/Mechanism	Direct genetic risk factors for disease susceptibility and severity (11)	Affect gene expression levels in relevant immune cells (19)
Pathway Involvement	Antigen presentation, Th17 pathway, immune response (14)	Regulation of gene transcription and cytokine signaling (19)
Reported Effect	Strongest known heritable risk HLA-B27; ERAP1 amplifies HLA-B27 risk (11)	Modulate expression in CD8+ T cells, TNF signaling, IL-6 pathway (19)

CD8+ T cells, Cluster of Differentiation 8 Positive T cells; eQTLs, Expression Quantitative Trait Loci; ERAP1, Endoplasmic Reticulum Aminopeptidase 1; HLA-B27, Human Leukocyte Antigen B27; IL23R, Interleukin-23 Receptor; IL6R, Interleukin-6 Receptor; QTLs, Quantitative Trait Loci; RUNX3, Runt-Related Transcription Factor 3; SNPs, Single Nucleotide Polymorphisms; TNFRSF1A, Tumor Necrosis Factor Receptor Superfamily Member 1A; TNFSF15, Tumor Necrosis Factor Superfamily Member 15.

## 6 Controversies and methodological challenges

### 6.1 Reproducibility and context dependency

eQTL effects are highly context-dependent, varying across tissues, cell types, and environmental conditions. This variability poses significant challenges for replicating findings across studies (24). Overcoming these hurdles necessitates the development of standardized protocols, comprehensive data collection, and the establishment of large-scale collaborative efforts to enhance reproducibility and interpretability.

### 6.2 Statistical power and multiple testing

Small sample sizes and the need for stringent multiple-testing corrections remain significant barriers in eQTL studies. These challenges can lead to reduced statistical power and the potential for false negatives. Recent advancements in statistical methodologies, including the application of Bayesian frameworks, are enhancing the robustness and reliability of eQTL analyses (25). Increasing sample sizes through consortia and leveraging meta-analyses can further address these limitations.

### 6.3 Integration of multi-omics data

Clinically comprehensive consideration of the genetics of diseases requires the simultaneous analysis of eQTL data with epigenomic, proteomic, and other omics datasets. However, this integration involves a plethora of methodological and computational challenges, including the necessity for complex tools that can handle high-dimensional data. Multi-omics approaches seem most promising for the development of integrative models of biological systems that link genetic variation to phenotypic outcomes (26).

### 6.4 Unraveling trans-eQTL networks

The functional roles and mechanisms of trans-eQTLs are still largely an unexplored territory. Such distally acting regulatory interactions are critical to understanding the complexities of gene networks and need thorough exploration (27). Advancing this field will provide insights into the systemic effects of genetic variants on gene expression across diverse cellular contexts.

### 6.5 Functional validation of eQTLs

The functional validation of eQTLs is crucial to confirm their regulatory effects and interpret the biological significance of genetic associations identified in GWAS. This process typically involves integrating *in vitro* experimental assays, CRISPR-Cas9 genome editing, reporter gene assays, and single-cell RNA sequencing (scRNA-seq) to experimentally verify the influence of candidate regulatory variants on gene expression (28, 29). For example, CRISPR interference (CRISPRi) and CRISPR activation (CRISPRa) technologies have been employed to silence or enhance specific enhancer or promoter regions encompassing eQTL variants, allowing direct assessment of their regulatory roles. Fulco et al. (2019) applied high-throughput CRISPR perturbation in T cells to validate enhancer–promoter interactions predicted by eQTL studies, confirming causal regulatory relationships in immune gene networks (30).

In parallel, single-cell RNA sequencing combined with genotype data enables the identification of cell-type–specific eQTLs and their functional consequences at unprecedented resolution. Van der Wijst et al. (2018) used this approach to map eQTLs in immune cell subsets, revealing how genetic variants differentially affect gene expression in specific cell types under both basal and stimulated conditions (20).

These experimental strategies have already been successfully applied in cardiovascular genomics and oncology to dissect the functional consequences of non-coding GWAS variants (31, 32). For instance, Musunuru et al. (2010) functionally validated a non-coding SNP at the SORT1 locus influencing cholesterol levels using CRISPR-modified hepatocyte models, establishing a mechanistic link between genetic variation and disease phenotype (31). Such approaches are increasingly vital for prioritizing candidate loci for drug development and for clarifying disease mechanisms. Moving forward, integrating CRISPR-based functional genomics with high-resolution transcriptomics and epigenomics will be essential for comprehensive eQTL validation in complex diseases like SpA.

### 6.6 Therapeutic implications

Understanding eQTL-regulated pathways opens new avenues for therapeutic innovation. For example, targeted modulatory effects on pathways (e.g., IL-23/IL-17 axis) or inhibition of ERAP1 activity is a novel therapeutic strategy (33). In addition, applications based on eQTL data that leverage personalized medicine approaches have the capacity to transform the treatment of SpA and other heterogeneous diseases by tailoring treatment to each person’s genotype.

## 7 Discussion

The incorporation of eQTL research to understand the pathogenesis of spondyloarthropathies (SpA) has brought new understanding to the role of genetic and molecular pathogenesis in complex inflammatory disorders. Completing the genetic relation between variants and gene expression alterations in disease backgrounds by eQTL studies has unraveled important signalling cascades, e.g., the IL-23/IL-17 axis, which is of central pathophysiological importance for SpA. Tissue-specific eQTL analyses have yielded information on local regulatory impact, but their generalizability from population to population and species to species is still an important limitation. Variations due to heterogeneous populations, environmental effects, and experimental approaches complicate the determination of eQTLs and emphasize the requirement for repeatability across large, heterogeneous cohorts (34).

The need for context in eQTL effects shows why we should use multi-omics approaches that bring everything together. When we combine eQTL data with information from transcriptomics, epigenomics, and proteomics, we can figure out the complex networks that control SpA. Take ATAC-seq data on chromatin accessibility or histone modification profiles as an example. These could help us understand how non-coding variants affect gene expression (35). On the other hand, including proteomics could link changes at the transcript level to what proteins do. This would help us learn more about SpA-specific pathways, like how ERAP1 processes and presents antigens.

Recent technological advances have opened new horizons for the functional validation of eQTLs. Single-cell RNA sequencing enables the dissection of cell-type–specific eQTL effects at unparalleled resolution, uncovering regulative mechanisms within clinically relevant tissues such as synovium and entheses. Furthermore, CRISPR-based gene editing offers precise tools for manipulating prospect eQTLs, facilitating the direct assessment of their roles in regulating gene expression and modulating unaffected responses (36). However, problems remain in the extension of such techniques to high-throughput applications and in the applicability of such techniques to tissue-specific situations. For SpA, obtaining sufficient quantities of synovial or entheseal cells from patients presents logistical and technical obstacles.

From a therapeutic perspective, eQTL research holds transformative potential in the era of precision medicine. Using patient-dependent eQTL profiles, clinicians may stratify patients according to their genetic and transcriptomic profiles. Such stratification may help to direct targeted therapy, i.e., biologics against signaling in the IL23R-mediated pathway or against ERAP1-associated pathways, thereby maximizing the therapeutic efficiency with reduced toxicity (37). In addition, these strategies have the potential to offer novel insights in the context of SpA treatment-resistant patients, which will be helpful in the development of new next-generation SpA therapies.

The successful execution of eQTL studies requires the coordinated collaboration of experts from multiple disciplines. Through interdisciplinary engagement, such projects can be effectively designed, implemented, and translated into meaningful biological and clinical insights. Also, the cooperation of genetic biologists, bioinformaticians, immunologists, and rheumatologists is vital for the implementation of the strong studies to come about. The introduction of advanced techniques in statistics, like those of eQTL prediction based on machine learning tools and the possibility for the availability of big data through large-scale collaborations such as GTEx or ENCODE, would be one of the main drivers of success (38). Besides the above actions, sharing the discovered eQTLs with the prognosis and the interventions used will be the key for translating end-patient genetic findings into the real-life world.

In conclusion, although difficulties remain, the incorporation of eQTL studies into SpA still promises a wide range of decoding disease mechanisms and discovery of new therapeutic targets. Overcoming the translational challenge from genetic findings to clinical practices will create a shortcut for more tailored and optimal management of spondyloarthropathies.
